# Growth Performance, Carcass Characteristics, and Blood Metabolites of Lambs Supplemented with a Polyherbal Mixture

**DOI:** 10.3390/ani11040955

**Published:** 2021-03-30

**Authors:** José Felipe Orzuna-Orzuna, Griselda Dorantes-Iturbide, Alejandro Lara-Bueno, Germán David Mendoza-Martínez, Luis Alberto Miranda-Romero, Pedro Abel Hernández-García

**Affiliations:** 1 Departamento de Zootecnia, Universidad Autónoma Chapingo, Chapingo CP 56230, Mexico; jforzuna@gmail.com (J.F.O.-O.); griseldi0993@gmail.com (G.D.-I.); albertomiranda@correo.chapingo.mx (L.A.M.-R.); 2 Departamento de Producción Agrícola y Animal, Unidad Xochimilco, Universidad Autónoma Metropolitana, Mexico City CP 04960, Mexico; gmendoza@correo.xoc.uam.mx; 3 Unidad Académica Amecameca, Universidad Autónoma del Estado de Mexico, Amecameca CP 56900, Mexico; pedro_abel@yahoo.com

**Keywords:** fattening lamb, meat quality, hematological profile, biochemical profile

## Abstract

**Simple Summary:**

Herbal products as feed supplements show beneficial effects on the productive performance and health of non-ruminants, but there is limited information about the effects in ruminants. The objective of this study was to evaluate the effects of a polyherbal mixture on growth performance, carcass characteristics, meat quality, and concentration of blood metabolites in lambs during the fattening period. Polyherbal mixture supplementation improved dry matter intake and increased the live weight of lambs without affecting carcass characteristics or meat quality. Polyherbal mixture supplementation was associated with lower blood creatinine concentration suggesting beneficial effects on the renal health condition of lambs. The results suggest that the use of herbal mixtures as additives in diets of finishing lambs can improve productivity without affecting carcass characteristics and meat quality.

**Abstract:**

The objective of this study was to determine the effects of the supplementation of a polyherbal mixture (HM) on the productive performance, carcass characteristics, meat quality, and the profile of blood metabolites of lambs fed a high-concentrate diet. Thirty-six male Pelibuey lambs (25.21 ± 0.96 kg BW) were housed in individual pens during a 56-day feeding period and were randomly assigned to four treatments: (1) Control (CON): Basal diet without HM; (2) HM1: CON + 1 g of HM kg^−1^ dry matter (DM); (3) HM2: CON + 2 g of HM kg^−1^ DM; and (4) HM3: CON + 3 g of HM kg^−1^ DM. Data were analyzed using the GLM (General Linear Model) procedure of statistical analysis system (SAS), and linear and quadratic effects were tested to evaluate the effects of the HM level. A quadratic increase was observed in the dry matter intake and in daily weight gain (*p* < 0.05) of lambs fed with HM2 and HM1, respectively. However, final body weight, body condition, carcass characteristics, and meat quality were similar among treatments (*p* > 0.05). It was observed a linear increase (*p* < 0.05) in the mean corpuscular hemoglobin concentration. Lymphocytes in blood from lambs supplemented with the HM1 diet increased and segmented neutrophils decreased compared to lambs receiving the CON treatment (*p* < 0.05). The concentration of uric acid in the blood had a linear increase (*p* < 0.05) and the serum creatinine level decreased (*p* < 0.05) as the HM dietary dose increased. In conclusion, dietary inclusion of 2 and 1 g of HM kg^−1^ of DM improves feed consumption and daily weight gain, respectively, without affecting carcass characteristics, meat quality, and health status on finishing lambs.

## 1. Introduction

Growth promoters have been widely used to increase the productivity of ruminants and non-ruminants. However, in several countries, the use of these products has been prohibited, which has led the industry and researchers to search for dietary supplementation with herbal additives as an alternative [1]. Some herbal products that contain phenols or flavonoids have shown promising effects when used as food additives for lambs and cattle [2,3]. Animunin Powder^®^ is a polyherbal mixture (HM) composed of parts from various plants such as *Solanum xanthocarpum* and *Hedychium spicatum*, which contain a high concentration of phenolic and flavonoid compounds [4,5].

Previous studies have shown that herbal products (7.8 and 16% kg^−1^ of DM) containing flavonoids stimulate muscle protein synthesis [6] and increase serum levels of the growth hormone in sheep [7]. In addition, these compounds have been successfully used to increase the duodenal flow of amino acids and microbial protein [8] and to reduce methane production in cattle [9]. Other phenol-containing products (10 g kg^−1^ of DM) have shown a positive impact on immune response, rate of rumen fermentation, and rumen microbial activity [10]. Diverse sources of phenolic compounds (3.2 mg d^−1^) have also been used to improve the quality of meat in lambs and goat kids [6,11]. The inclusion of phenols and flavonoids in the diet (3.4 and 10 g kg^−1^ of DM) could increase weight gain in lambs through changes in the rumen microbiome [12]. Nevertheless, the effects of both secondary metabolites on the animal response appear to be dependent on the dose and the source from which they are derived. [2,6,7,13]. Due to the beneficial effects of herbal products and their secondary metabolites, it has been hypothesized that supplementation with HM as a source of phenols and flavonoids can contribute to improving the productivity of the lambs during the final fattening period without affecting the quality of the meat or the health of the animals. However, there is little information on the effects of herbal products containing phenols and flavonoids on the productivity of fattening lambs. The objective of this study was to evaluate the effect of increased doses of HM containing phenols and flavonoids on the productive performance, carcass characteristics, meat quality, and concentration of blood metabolites of lambs fed high-concentrate diets.

## 2. Materials and Methods

### 2.1. Location

The experiment was conducted at the Teaching and Research Unit of Small Ruminants located at the Experimental Farm of the Universidad Autónoma Chapingo, Mexico, located at the 19°22′ north latitude 98°35′ west longitude, with an altitude of 2250 m. The climate is temperate subhumid with rain during the summer and dry during the winter, with average annual temperature and precipitation of 15.2 °C and 665 mm, respectively [14]. The care and handling procedures for the lambs were carried out following the guidelines of the Official Mexican Standard (NOM-062-ZOO-1995).

### 2.2. Diet Composition and Management

The lambs were fed a finishing diet comprised of 19.4% oat straw, 24.1% ground sorghum, 8.1% soybean meal, 30.3% ground corn, 7.1% wheat bran, 7.4% corn gluten, 2.3% bypass fat, 0.3% calcium carbonate, 0.5% salt, 0.5% vitamin and mineral supplement (DM basis). The nutrient composition of the basal diet was 15.69% crude protein, 2.64% ether extract, 26.04% neutral detergent fiber, 13.75% acid detergent fiber, 5.52% ash, and 2.8 Mcal of metabolizable energy (DM basis). HM was fed to the lambs through diets formulated to have weight gains of 300 g d^−1^ [15]. The HM was mixed with the few hundreds of corn grams then they were mixed the total diets.

The feed was provided at 08:00 and 16:00 h, and the drinking water was supplied ad libitum. The lambs were weighed after 12 h post fasting on 2 consecutive days, at the beginning (day 0 and 1), and weighed at the end of the experimental period (day 55 and 56). The variables measured were: Initial body weight (IBW) measured after the adaptation period, final body weight (FBW), dry matter intake (DMI), and the daily weight gain (DWG) calculated as (FBW − IBW)/56. The body condition scoring (BCS) was determined on day 56 of the experiment using a 5 category scale (1 to 5), assigning BCS = 1 to an emaciated animal, BCS = 3 to an animal in average body condition, and BCS = 5 to an obese animal [16].

### 2.3. Polyherbal Mixture Characteristics

The HM used was Animunin Powder^®^ (Nuproxa S. de RL. de CV. Querétaro, México), which was a labeled commercial herbal formula that came from the same batch, composed of plant parts from *Solanum xanthocarpum*, *Hedychium spicatum*, *Curcuma longa*, *Piper longum*, and *Ocimum sanctum*. *S. xanthocarpum*, which contains flavonoids and phenols with antioxidant activity [4]; *H. spicatum* contains phenolic and flavonoid compounds with antimicrobial, anti-inflammatory, and antioxidant activities [5]; *C. longa* contains curcumin, a polyphenolic substance with antioxidant and anti-inflammatory effects [17]; *P. longum* contains phenols and flavonoids such as quercetin and naringenin [18]; and *O. sanctum* contains phenols with antifungal activity [19].

### 2.4. Experimental Design

**Figure 1:**
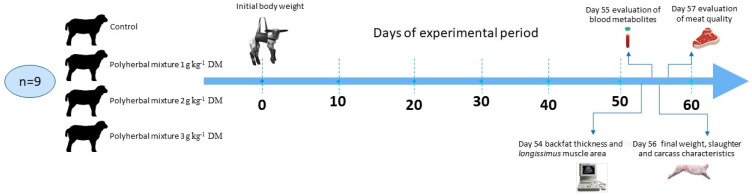
Completely randomized design and sampling times of lambs supplemented with a polyherbal mixture during the final fattening period; n = 9 indicate the number of animals sampled in each treatment; DM = dry matter.

Thirty-six male Pelibuey lambs (25.2 ± 0.9 kg BW, 4–5 months old) were randomly distributed to 4 treatments: (1) Basal diet without HM (CON); (2) HM1, CON + 1 g of HM kg^−1^ dry matter (DM); (3) HM2, CON + 2 g of HM kg^−1^ DM; and 4) HM3, CON + 3 g of HM kg^−1^ DM. The lambs were placed in individual pens (2.6 m × 0.8 m) equipped with individual feeders and automatic drinkers. Prior to the start of the experimental phase, the lambs were dewormed through an oral administration of Koptisin ovine^®^ (Chinoin Labs, México City, México, 10 mg kg^−1^ BW), and vaccinated against *Pasteurella* and *Clostridium* (Bobact^®^ 8 MSD-Merck, Kenilworth, NJ, USA, 2.5 mL lamb^−1^). The lambs had an adaptation period to the basal diet of 14 days, and the experimental phase lasted 56 days. Figure 1 shows the experimental procedure.

### 2.5. Sampling and Analyses of Feeds

Samples of feed provided and rejected were collected daily to determine the chemical composition. Prior to the analysis, the food samples were dried at 55° C in a forced-air oven and then ground in a Wiley mill (model 4, Arthur Thomas Co., Philadelphia, PA, USA). The variables determined were: Dry matter, ether extract, crude protein, and ash [20]. Neutral detergent fiber and acid detergent fiber were determined using the procedures described by Van Soest et al. [21].

### 2.6. Carcass Characteristics and Meat Quality

The backfat thickness (BFT) and the *longissimus* muscle area (LMA) located between the 12th and 13th ribs of the lamb were measured on day 54 of the experiment using a Sonovet 600 (Medison, Inc., Cypress, CA, USA) with a 7.5 Mhz transducer [22]. After the last weighing (day 56 of the experiment), the lambs were fasted for 16 h before being slaughtered. The slaughter process was conducted in a commercial slaughterhouse in accordance with standard procedures of the Official Mexican Standard (NOM-033-SAG/ZOO-2014). Immediately after the slaughter, the hot carcass weight was registered (HCW), and subsequently, the carcasses were left to repose at 4 °C for 24 h to register the cold carcass weight (CCW). The hot carcass yield (HCY) and the cold carcass yield (CCY) were determined through: HCY = (HCW/FBW) × 100 and CCY = (CCW/FBW) × 100, as it was described by Zimerman et al. [23]. The body morphometry, external length of the carcass (ELC), internal length of the carcass (ILC), length of the leg (LL), perimeter of the leg (PL), and the carcass compactness index (CCI), were obtained based on the methodology described by Yañez et al. [24], where: CCI = CCW/ILC, in kg cm^−1^. In addition, the head, legs, skin, rumen (full and empty), liver, spleen, kidneys, heart, lungs, small intestine, and large intestine were each weighed separately.

Muscle samples were collected from the cold carcass *longissimus dorsi* (approximately 400 g) and frozen at −20 °C for later analysis of meat quality. The meat color was measured in cuts of the muscle 24 h after slaughter using a spectrophotometer Minolta CM-2006d (modelo Konica, Minolta Holdings Inc., Osaka, Japan). Lightness (L*), redness (a*), and yellowness (b*) were evaluated as attributes of meat quality using the procedure proposed by Ripoll et al. [25]. With the values of a* and b*, the Croma (C*) and Hue (H*) indexes were calculated using the equations: Croma = (a*2 + b*2)^0.5^ and Hue = tan^−1^(b*/a*) × 57.29 both expressed in degrees [25]. The color coordinate values were calculated using the average of 3-color measurements for each sample. The pH of the meat was obtained following the procedure described by Negrete et al. [26]. This was measured 3 times in 3 g of muscle *longissimus dorsi* homogenized with deionized water (20 mL) with a blender Waring 51BL32 (model 700, Torrington, CT, USA), using a pH meter Hanna^®^ (Model HI 98127, Waterproof Tester, EE. UU.). Previous to the proximate analysis of the meat, the samples were defrosted at 4 °C for 24 h. The subcutaneous fat and connective tissue were removed from the muscle with a scalpel, and the meat was ground and then homogenized for 5 min in a blender. Subsequently, 180 g of sample was taken in triplicate to determine protein, fat, moisture, and collagen (g kg^−1^) using a near-infrared spectrophotometer FOSS FoodScan™, as described by Anderson [27].

### 2.7. Blood Metabolites

On day 55 of the experimental phase, blood samples were taken from the jugular vein of the lambs, before the morning feeding (08:00 h) with the purpose of determining hematological and biochemical parameters. For determining the hematological parameters, the methodology described by Ayala et al. was followed [28]. For this, 5 mL of blood was extracted from each lamb in tubes BD Vacutainer^®^ K2 EDTA with anticoagulant. An additional 5 mL of blood was collected in tubes BD Vacutainer^®^ without anticoagulant to obtain blood serum. The samples were stored at 4 °C to measure the red blood cells, hematocrit, platelets, hemoglobin, and leukocyte, with an automated hematology analyzer (Sysmex, XS-1000i™, Kobe, Japan). The samples without anticoagulant were centrifuged (centrifuge Sigma, 2–16k, Osterode am Harz, Germany) at 3500 rpm for 20 min, and the serum obtained was stored in tubes Eppendorf at −20 °C. The concentration of blood metabolites: Cholesterol, glucose, total protein, albumin, globulin, creatinine, bilirubin, urea, uric acid, alkaline phosphatase, and lactate dehydrogenase, were determined using a blood auto-analyzer (EasyVet, KontroLab ES-300, Michoacán, México) and using Spinreact kits (Barcelona, Spain).

### 2.8. Statistical Analysis

First, the normality test was performed on all variables using the SAS UNIVARIATE procedure [29]. Afterward, the data were analyzed according to the SAS GLM (General Linear Model) procedure [29]. Both linear and quadratic orthogonal polynomials were used to evaluate the effects of the level of the polyherbal mixture in the diet on each of the target variables. Initially, IBW was included as a co-variable to adjust the FBW, DWG, and DMI variables. However, this co-variable was removed from the model because it was not significant (*p* > 0.05). Significant differences were considered when *p* ≤ 0.05, and a trend was considered when *p* >0.05 but ≤0.10.

## 3. Results

### 3.1. Productive Performance

FBW showed a tendency of quadratic increase (*p* = 0.07), and the lambs that consumed the HM1 diet presented a higher body weight at the end of the experimental trial compared to the lambs assigned to the other treatments (Table 1). DMI, DWG, and TWG also showed quadratic increases (*p* ≤ 0.05). The lambs assigned to the control group (CON) and to the HM3 treatment had lower averages (*p* = 0.05) compared to the lambs that consumed the HM1 and HM2 diet. On the other hand, BCS showed a tendency of quadratic increase (*p* = 0.10), and the lambs that were supplemented with HM1 performed higher than the lambs fed with the other diets. However, the feed conversion ratio was not affected by the level of HM added to the diet (*p* = 0.15).

### 3.2. Carcass Characteristics and Non-Carcass Components

The carcass characteristics and the body morphometry are shown in Table 2. The hot carcass weight, the cold carcass weight, the hot carcass yield, the cold carcass yield, losses due to carcass cooling, the backfat thickness, and the muscle area *longissimus dorsi*, were similar in the lambs of all treatments (*p* > 0.05). Similarly, the external length of the carcass and the internal length of the carcass, the perimeter and the length of the leg, and the carcass compactness index were also not affected by the addition of HM to the diet (*p* > 0.05).

On the other hand, a tendency of linear increase (*p* = 0.08) was observed in the average weight of the liver, and a tendency of quadratic increase (*p* = 0.09) in the weight of the heart after slaughter, while for other internal organs (rumen, small intestine, large intestine, lung, kidney, spleen), skin, head, and feet, the weights were similar (*p* > 0.05) in the lambs across all treatments (Table 3).

### 3.3. Meat Quality

There were no significant effects (*p* > 0.05) and no trends (*p* > 0.10) of the supplementation HM on the chemical composition (protein, fat, moisture, and collagen), pH, and color of the meat (L*, a*, b*, Chroma, and Hue°; Table 4).

### 3.4. Hematological Variables

The results of the blood metabolite analysis on lambs supplemented with HM are reported in Table 5. Most blood components were not affected by the level of HM in the diet. However, a positive linear increase (*p* = 0.01) was observed in the mean corpuscular hemoglobin concentration as the dose of HM increased. In contrast, a tendency of linear decrease in basophil concentration (*p* = 0.06) was observed. Lymphocytes in blood from lambs supplemented with the HM1 diet increased by 35% (*p* = 0.004), and segmented neutrophils decreased by 13% compared to lambs receiving the CON treatment (*p* = 0.001).

### 3.5. Blood Biochemistry

Table 6 shows the effects of increasing doses of HM on the serum biochemical parameters. Apart from urea, uric acid, and creatinine, all other metabolites were not affected by the level of HM in the diet (*p* >0.05). Urea concentration showed a tendency of quadratic increase (*p* = 0.09), while uric acid concentration increased linearly (*p* = 0.04) and creatinine concentration decreased linearly (*p* = 0.02) as the dose of MH in the diet increased.

## 4. Discussion

In all treatments, lambs showed DWG and FBW within the range reported in the literature for hair lambs of similar age fattened on high concentrate rations [30]. The lambs fed with the HM1 diet were 6% heavier at the end of fattening compared to lambs fed with the CON diet. In addition, DMI was 11% higher in lambs fed with HM2 compared to lambs assigned to CON. In a similar study, Razo et al. [31] examined the effects of HM (0, 1, 2, and 3 g kg^−1^ DM for 60 days) based on *Withania somnifera*, *Ocimum tenuiflorum*, *Tinospora cordifolia*, and *Emblica officinalis* containing polyphenols and flavonoids, on lambs fed high-concentrate diets. In their investigation, lambs supplemented with low doses of polyphenols and flavonoids had higher DWG, DMI, and FBW compared to the other treatments. However, as the dose of polyphenols and flavonoids increased, the growth performance worsened, similar to the results observed in our study. In another study by Lobo et al. [2], the effects of supplementing lambs were evaluated using leaf extracts from *Illex paraguariensis* (0, 1, 2, and 4% for 53 days) containing phenolic compounds. It was observed that DWG, DMI, and FBW increased at doses up to 2% of the extract in the diet, however, these decreased at doses of 4%. These results suggest that the effects of phenols and flavonoids can improve the productive performance at low doses, but at high doses, they could affect the growth rate, probably due to toxic effects. The higher DMI observed in lambs that consumed HM1 was potentially associated with higher digestibility, as previously reported by Ma et al. [32] in lambs supplemented with 0.25 mg d^−1^ of resveratrol (a natural polyphenol). The inclusion of flavonoids in the diet can reduce the concentration of propionate in the rumen by changes in the fermentation carried out by the rumen microbiome [3,10]. Additionally, supplementation with flavonoids has the potential to modify gene expression involved in the regulation of DMI [3]. Similar effects from the consumption of flavonoids seen in our study would partially explain a higher DMI in the lambs supplemented with 1 and 2 g of HM kg^−1^ DM.

On the other hand, Du et al. [12] reported that supplementation with flavonoids extracted from the plant *Allium mongolicum* increased DWG by increasing the presence of bacteria (*Tenericutes* and *Mollicutes*) positively correlated with DWG in the rumen microbiome. It is suggested that the presence of flavonoids in the diet consumed by lambs stimulates the protein synthesis in muscle and inhibits proteolysis [6], also increasing the duodenal flux of amino acids and microbial protein [8]. In addition, a recent study in beef cattle showed that a 0.04% flavonoid dietary supplementation extracted from *Citrus aurantium* improves rumen health [3,8]. This results in higher absorption of propionate through the rumen wall—a higher metabolic availability of nitrogenous compounds—which, in our case, explains the higher DWG in lambs supplemented with HM.

Results from other studies suggest that the addition of phenolic and flavonoid compounds in the diet acts by increasing the serum levels of growth hormone [7], reducing the production of enteric methane [9], increasing digestibility [31], and improving energy utilization [33]. These findings could explain the increases in DMI and DWG observed in lambs that consumed HM in the present study

Feed conversion ratio and BCS were similar for all treatments. These results are congruent with the findings of Lobo et al. [2], who used leaf extracts of *Illex paraguariensis* (0, 1, 2, and 4% for 53 days) containing phenolic compounds to feed lambs. In their study, CA and BCS were not affected by the supplementation of the extract in the diet. However, it was observed that TWG and carcass weight were higher in lambs supplemented with 2% of the extract. Additionally, Odhaib et al. [10] also observed no differences in CA of lambs fed with high-concentrate diets supplemented with *Rosmarinus officinalis* leaves, *Nigella sativa* seeds and a combination of both plants, containing 12.35, 19.08, and 34.86 mg of total polyphenols kg^−1^ DM, respectively. Although DWG increased significantly in lambs that consumed HM, in our study, DMI also increased, which explains the absence of significant changes in CA.

Regarding the carcass characteristics, the results of the present study agree with those reported by Simitzis et al. [34] in lambs that consumed diets with a high proportion of concentrate, supplemented with a pure flavonoid (hesperidin) at dietary concentrations of 1500 and 3000 mg kg^−1^ of feed for 35 days. In their study HCW, CCW, and HCY were similar among treatments, perhaps as a consequence of the low impact of flavonoids supplementation on the FBW of the lambs and on the nutritional composition of the diet consumed, similar to what was observed in this study. Cimmino et al. [11] also observed no differences in HCW, CCW, HCY, and CCY of goat kids fed with high-concentrate diets and supplemented with polyphenols (3.2 mg d^−1^ for 78 days) taken from residual water after the olive oil extraction. However, polyphenols had positive effects on the fatty acid profile of the meat. The similarity of BFT in carcasses across all treatments could also partially explain the lack of changes in the carcass performance observed in the present study.

BFT and LMA values were also similar among treatments. Similar results were reported by Valero et al. [35] in bulls supplemented with 0.162 g d^−1^ of flavonoids extracted from propolis. On the contrary, Lobo et al. [2] used incremental doses (0, 1, 2, and 4% for 53 days) of extracts from *Illex paraguariensis* leaves, which contain phenols, in a 60% concentrate diet. Their results showed that BFT decreased as the extract dose increased. However, LMA was higher in lambs receiving the 2% extract dose, resulting in a higher lean muscle production, thus suggesting that the effects of phenols are dose-dependent. The mechanism of action of phenols and flavonoids on lipogenesis has not been studied in lambs. However, in beef cattle fed with high concentrate diets, the inclusion of isoflavone in the diet increased BFT, and it enhanced the synthesis of triglycerides on subcutaneous adipocytes by changing the differential expression of genes involved in lipid metabolism [36]. In the present study, BFT was not affected by the inclusion of HM despite the fact that it contained flavonoids, which indicates that the source of flavonoids has an influence on the changes in BFT. Although genotype, age, sex, and weight of lambs show positive correlations with carcass fat deposition [37] and with carcass physical and chemical characteristics [38,39], the homogeneity of these characteristics in the lambs used in the treatments partially explains the absence of changes in BFT and LMA.

Similar results on body morphometry and carcass compactness index were previously reported by Lobo et al. [2] in lambs supplemented with extracts (0, 1, 2, and 4%) from *Illex paraguariensis* leaves, containing phenolic compounds. However, there is limited information about the effects of phenolic and flavonoid compounds derived from herbal mixtures on morphometric measurements of ruminant carcasses. Perhaps, slaughter weight, cold carcass weight, muscle mass, and adipose tissue deposition are related to these variables [24].

Regarding the organs, similar results were observed by Simitzis et al. [13,34] in lambs fed with high concentrate diets enriched with 2500 mg of purified flavonoids (hesperidin or naringenin) and with 1500 and 3000 mg of hesperidin kg^−1^, respectively. Information about the effects of phenol and flavonoid consumption on the size and weight of internal organs of lambs is still limited, which complicates the explanation of the results observed in this and other studies. However, according to Riley et al. [40], differences in weight and size of internal organs in ovines are influenced by genotype, sex, and age of the animals, and by the dietary restrictions [41]. In addition, the tendency for liver and heart weights to increase in lambs supplemented with HM could be explained by their higher feed consumption [42].

The average values of the chemical composition of meat are in the range reported in the literature for lambs fed high-concentrate diets [30]. Similar results were previously reported by Qin et al. [6] on lambs fed with pomace (0, 7.8, and 16% for 80 days) obtained from the *Hippophae rhamnoides* fruits, containing 0.69 and 1.02% of flavonoids, respectively. In their study, the dose of flavonoids did not affect the moisture, protein, and ash content of the meat. However, the high dose of flavonoids increased the fat content, which according to the authors, could result in a juicier and softer meat. Cimmino et al. [11] also observed higher fat content, but similar moisture content, protein, ash, and collagen in meat from goat kids fed with high-concentrate diets supplemented with polyphenol extracts. (3.2 mg d^−1^ for 78 days) taken from residual water after the olive oil extraction. These results suggest that the effects of phenols and flavonoids on the composition in lamb meat may be dependent on the dose, the botanical origin, and the duration of the experimental phase. On the other hand, considering that the chemical composition of meat varies according to the feeding regime [43] and by the slaughter weight [39], in our study, it is likely that the lack of effect on the chemical composition of meat was because HM had minimal effect on the nutritional composition and the FBW of the lambs.

The pH value of meat was similar among treatments but only the pH value of meat from lambs fed with HM was in the considered normal range between 5.5 and 5.8 [44]. These results suggest that supplementation with HM during the final fattening period could improve the meat quality compared to the meat of lambs not supplemented with HM. Our findings are largely congruent with the results of Qin et al. [6] in lambs supplemented with incremental levels (7.8 and 16% for 80 days) of pomace from *Hippophae rhamnoides* fruits, containing 0.69 and 1.02% of flavonoids, respectively. In addition, with the results of Simitzis et al. [13] in lambs supplemented with hesperidin or naringenin at dietary feed concentrations of 2500 mg kg^−1^; and results of Cimmino et al. [11] in goat kids supplemented with 3.2 mg d^−1^ of polyphenol extracts taken from residual water after the olive oil extraction. A pH value below 5.8 is important for the preservation of meat during storage as it has a bacteriostatic effect, while higher pH values favor the growth of proteolytic microorganisms [45]. This indicates that supplementation with HM during the final fattening period can increase the shelf life of lamb meat.

Additionally, in the present study, there were no effects on meat color attributes (L*, a*, b*, Chroma, and Hue°) associated with the inclusion of HM in the diet. Similar results were previously reported by Qin et al. [6] on meat from lambs fed with pomace from *Hippophae rhamnoides* fruits in the diet (7.8 and 16% for 80 days), containing 0.69 and 1.02% of flavonoids, respectively, and by Simitzis et al. [13] in lambs supplemented with hesperidin or naringenin at dietary concentrations of 2500 mg kg^−1^ of feed for 35 days. In addition, Muela et al. [46] did not observe changes in meat color in lambs supplemented with a commercial extract (150 mg kg^−1^ DM for 40 days) taken from whole fruits of *Citrus paradisi*, *Citrus aurantium bergamia*, *Citrus sinensis*, and *Citrus reticulata*, plants, containing 3.5% polyphenols and 0.8% bioflavonoids (naringenin, quercetin and rutin). These results suggest that the effects of phenolic and flavonoid compounds on the color of lamb meat are not dependent on the dose, the botanical origin, or the period of administration.

Color is an important attribute of meat quality because it is the first aspect that attracts consumers when choosing fresh meat [11]. Color stability depends on the oxidation of myoglobin and the formation and accumulation of metamyoglobin [11]. However, several studies [11,46] have reported that the inclusion of polyphenols and flavonoids in the diet does not affect myoglobin, oxymyoglobin, and metamyoglobin contents in lamb meat, which would explain the absence of changes in meat color attributes observed in the lambs that were fed with HM in the present study.

The evaluation and validation of a new feed additive require the assessment of the health status of the animals after its consumption. In our study, the hematological parameters of lambs were similar among treatments and showed values within the normal physiological range [47]. In a similar study, Razo et al. [31] were investigated the effects of supplementation with a polyherbal mixture (0, 1, 2, and 3 g kg^−1^ DM for 60 days) containing polyphenols and flavonoids on blood metabolites in lambs that were fed high-concentrate diets. In their study, they observed the highest platelet concentration in lambs consuming 1 g of HM, however, platelets decreased as the dose of HM increased, thus indicating that phenols and flavonoids can stimulate the immune response at low dose, but at high doses they may depress the immune system. On the other hand, Morsy et al. [48] evaluated the effects of supplementation with red propolis (3 g d^−1^ for 21 days) containing 43% of isoflavonoids in pregnant ewes fed with 50% of roughage in the diet and observed that the presence of isoflavonoids improved the total leucocyte concentration, total protein, and globulin in the blood. These results suggest that flavonoids can stimulate the immune system regardless of the physiological stage of the animal or the source of the metabolites, even when flavonoids are administered for short periods.

In our study, it was observed a quadratic reduction in the concentration of segmented neutrophils in lambs that consumed 1 and 2 g of HM. Similar results were reported by Molosse et al. [32] in suckling lambs supplemented with curcumin, and by Odhaib et al. [10], where lambs were fed high-concentrate diets supplemented with 1% of *Rosmarinus officinalis* leaves, *Nigella sativa* seeds, or a combination of both plants containing 12.35, 19.08, and 34.86 mg of total polyphenols kg^−1^ DM, respectively. Their results also showed that polyphenols improved the immune response without affecting hematological parameters or blood biochemistry in all treatments. Generally, the reduction of neutrophils occurs in the presence of bacterial infections, however, there are other factors that can decrease the number of neutrophils in healthy individuals [49].

The blood concentration of lymphocytes often increases in animals under stress, excitement, and fear [50] or during bacterial infections [47]. Lobo et al. [2] observed no changes in the concentration of lymphocytes in lambs supplemented with 1, 2, and 4% extract of *Illex paraguariensis* leaves as a source of phenols. On the other hand, in the present study, the concentration of lymphocytes increased in lambs supplemented with HM. However, lambs supplemented with HM showed no signs of disease during the experimental phase and even had better productive performance compared to lambs assigned to the control treatment.

According to Braun et al. [51], biochemical parameters in ovines are mainly used for the diagnosis of liver, muscle, and nutritional disorders. In the present study, the concentration of these blood metabolites was in the normal range for ovines [52], suggesting that the HM used did not cause liver, muscle, or nutritional disorders in the lambs. Similar results were previously reported by Lobo et al. [2] on lambs supplemented with 1, 2, and 4% extract of *Illex paraguariensis* leaves containing phenols; by Qin et al. [6] on lambs supplemented with 7.8 and 16% of pomace from *Hippophae rhamnoides* fruits, containing 0.69 and 1.02% of flavonoids; and by Razo et al. [31], where lambs were supplemented with incremental doses (0, 1, 2, and 3 g kg^−1^ DM for 60 days) of a polyherbal mixture of Indian plants containing polyphenols and flavonoids, and observed that the blood chemistry values of all treatments were within the ranges reported as normal for ovines.

Regarding serum uric acid and urea concentrations, similar results were previously reported by Zhong et al. [53] in lambs supplemented with polyphenols extracted from the green tea plant at concentrations of 2, 4, and 6 g kg^−1^ of feed for 56 days, and by Razo et al. [31], in lambs supplemented with a polyherbal mixture (1, 2, and 3 g kg^−1^ DM for 60 days) containing polyphenols and flavonoids. Their results showed that uric acid rose as the dose of polyphenols and flavonoids increased, however, the urea was similar among treatments. It has also been reported an increased amino acid and microbial protein duodenal flux in ruminants supplemented with flavonoids [8], which would partially explain the increase in urea and serum uric acid in lambs supplemented with HM.

In our study, lambs supplemented with HM showed low serum creatinine levels, but these were in the normal range for healthy ovine [52], while concentrations of that blood metabolite in the lambs assigned to the control group were up to 27% above the normal range. By contrast, Lobo et al. [2] reported an increase of serum creatinine in lambs as the dietary level of phenolic compounds was increased. On the other hand, it is known that serum creatinine increases in cases of chronic and acute renal failure [54], which suggests that the HM used in the present study did not affect renal health.

There is limited information about the use of herbal mixtures and their effects on the mineral status of ruminants. Calcium and phosphorus in blood serum are valuable indicators of the nutritional status of animals due to the low variability of its concentration in the blood [55]. The serum concentrations of calcium and phosphorus in our study were similar among treatments (*p* > 0.05) and were in the normal range for ovines [53], indicating that the consumption of HM did not affect the mineral balance in the diet or the nutritional status of the lambs. However, in a similar study, Razo et al. [31] investigated the effects of supplementation with a polyherbal mixture (0, 1, 2, and 3 g kg^−1^ DM for 60 days) containing polyphenols and flavonoids on blood metabolites of lambs fed high-concentrate diets. In that study, serum calcium was also not affected by the dose of polyphenols and flavonoids, however, serum phosphorus concentration increased as the dose of polyphenols and flavonoids increased. These results suggest that the effects of HM containing polyphenols and flavonoids on the mineral status of lambs depend on the dose used and the botanical origin.

## 5. Conclusions

The results of this study indicate that the inclusion of 2 and 1 g of HM kg^−1^ DM in the diet improves the feed consumption and weight gain, respectively, without affecting the carcass characteristics, meat quality, and health status of the lambs. HM Animunin^®^ can be used to improve the productivity of lambs fed high-concentrate diets. However, further research is required to test the effects of other doses of this HM in ovines at different concentrate proportions, experimental periods, and physiological stages.

## Figures and Tables

**Table 1:** Growth performance of lambs supplemented with a polyherbal mixture ^1^ during the final fattening period.

Parameter	Treatment				EEM	*p*-Value	
	CON	HM1	HM2	HM3		Linear	Quadratic
Initial body weight, kg	25.35	25.49	25.25	24.77	0.96	0.65	0.75
Final body weight, kg	36.80 *	39.29 *	38.33	36.63	1.15	0.77	0.07
Dry matter intake (DMI), kg d^−1^	0.965 ^b^	1.052 ^ab^	1.071 ^a^	0.999 ^ab^	0.04	0.48	0.04
Daily weight gain (DWG), kg d^−1^	0.204 ^b^	0.247 ^a^	0.233 ^ab^	0.207 ^ab^	0.02	0.89	0.05
Total weight gain (TWG), kg	11.45 ^b^	13.80 ^a^	13.07 ^ab^	11.85 ^ab^	0.89	0.89	0.05
Feed conversion ratio, DMI/DWG	4.91	4.33	4.66	4.99	0.27	0.77	0.15
Body condition scoring (BCS), 1 to 5	2.78 *	3.33 *	3.00	2.88	0.19	0.99	0.10

^1^ Animunin Powder^®^ based on *Solanum xanthocarpum*, *Hedychium spicatum*, *Curcuma longa*, *Piper longum* and *Ocimum sanctum*. CON—basal diet without polyherbal mixture (HM); HM1—basal diet + 1 g of HM kg^−1^ of DM; HM2—basal diet + 2 g of HM kg^−1^ of DM; HM3—basal diet + 3 g of HM kg^−1^ of DM. EEM—standard error of the treatment means; ^a,b^—means within a row with different subscripts differ when *p* ≤ 0.05; *—indicates a tendency.

**Table 2:** Carcass characteristics of lambs supplemented with a polyherbal mixture ^1^ during the final fattening period.

Parameter	Treatment				EEM	*p*-Value	
	CON	HM1	HM2	HM3		Linear	Quadratic
Hot carcass weight, kg	18.09	19.21	18.48	18.10	0.56	0.93	0.21
Cold carcass weight, kg	17.41	18.58	17.75	17.40	0.54	0.90	0.19
Hot carcass yield, %	49.17	48.89	48.23	49.43	0.69	0.97	0.29
Cold carcass yield, %	47.32	47.30	46.33	47.50	0.62	0.95	0.31
Losses due to carcass cooling, %	3.72	3.24	3.93	3.70	0.35	0.68	0.72
Backfat thickness, mm	2.89	3.00	3.00	2.89	0.08	0.99	0.17
Muscle area *longissimus dorsi*, cm^2^	10.57	10.84	10.44	10.63	0.40	0.89	0.91
External length of the carcass, cm	58.33	58.00	60.11	59.55	0.94	0.18	0.91
Internal length of the carcass, cm	56.88	56.67	57.89	57.11	0.82	0.61	0.74
Perimeter of the leg, cm	39.89	39.22	40.22	39.66	1.04	0.96	0.92
Length of the leg, cm	33.55	33.44	34.88	34.88	0.77	0.12	0.94
Compactness index, kg cm^−1^	0.31	0.34	0.32	0.32	0.01	0.68	0.22

^1^ Animunin Powder^®^ based on *Solanum xanthocarpum*, *Hedychium spicatum*, *Curcuma longa*, *Piper longum* and *Ocimum sanctum*. CON—basal diet without polyherbal mixture (HM); HM1—basal diet + 1 g of HM kg^−1^ of DM; HM2—basal diet + 2 g of HM kg^−1^ of DM; HM3—basal diet + 3 g of HM kg^−1^ of DM. EEM—standard error of the treatment means.

**Table 3:** Organ weights of lambs supplemented with a polyherbal mixture ^1^ during the final fattening period.

Parameter	Treatment				EEM	*p*-Value	
	CON	HM1	HM2	HM3		Linear	Quadratic
Rumen (full), kg	3.816	4.095	3.972	3.579	0.205	0.37	0.11
Rumen (empty), kg	1.397	1.368	1.633	1.585	0.113	0.11	0.93
Small intestine (empty), kg	0.701	0.767	0.677	0.698	0.053	0.69	0.68
Large intestine (empty), kg	0.780	0.855	0.890	0.886	0.067	0.25	0.56
Lungs and Trachea, kg	0.652	0.677	0.643	0.657	0.028	0.88	0.83
Heart, kg	0.157	0.191 *	0.157	0.152 *	0.011	0.35	0.09
Liver, kg	0.783 *	0.808	0.822	0.887 *	0.040	0.08	0.62
Kidneys, kg	0.474	0.473	0.475	0.481	0.046	0.90	0.95
Spleen, kg	0.087	0.078	0.070	0.078	0.006	0.21	0.16
Skin, kg	2.941	3.160	3.121	2.917	0.193	0.90	0.28
Head, kg	1.771	1.870	1.869	1.792	0.069	0.84	0.21
Feet, kg	0.837	0.863	0.856	0.861	0.034	0.68	0.77

^1^ Animunin Powder^®^ based on *Solanum xanthocarpum*, *Hedychium spicatum*, *Curcuma longa*, *Piper longum* and *Ocimum sanctum*. CON—basal diet without polyherbal mixture (HM); HM1—basal diet + 1 g of HM kg^−1^ of DM; HM2—basal diet + 2 g of HM kg^−1^ of DM; HM3—basal diet + 3 g of HM kg^−1^ of DM. EEM—standard error of the treatment means; *—indicates a tendency.

**Table 4:** Meat characteristics of lambs supplemented with a polyherbal mixture ^1^ during the final fattening period.

Parameter	Treatment				EEM	*p*-Value	
	CON	HM1	HM2	HM3		Linear	Quadratic
Protein, g kg^−1^	210.55	210.11	209.55	211.07	2.51	0.94	0.71
Fat, g kg^−1^	105.22	102.55	102.01	100.44	4.45	0.46	0.90
Moisture, g kg^−1^	670.77	672.66	676.66	672.78	4.82	0.64	0.55
Collagen, g kg^−1^	21.33	20.55	20.67	21.56	0.89	0.85	0.36
pH	6.04	5.73	5.87	5.87	0.11	0.45	0.15
Lightness (L*)	40.28	40.47	39.45	40.18	0.73	0.68	0.71
Redness (a*)	15.54	15.27	14.60	15.20	0.31	0.23	0.17
Yellowness (b*)	13.67	14.14	13.18	13.63	0.34	0.48	0.97
Croma	20.70	20.82	19.70	20.43	0.38	0.26	0.42
Hue°	41.33	42.78	41.99	41.92	0.74	0.77	0.31

^1^ Animunin Powder^®^ based on *Solanum xanthocarpum*, *Hedychium spicatum*, *Curcuma longa*, *Piper longum* and *Ocimum sanctum*. CON—basal diet without polyherbal mixture (HM); HM1—basal diet + 1 g of HM kg^−1^ of DM; HM2—basal diet + 2 g of HM kg^−1^ of DM; HM3—basal diet + 3 g of HM kg^−1^ of DM. EEM—standard error of the treatment means.

**Table 5:** Hematological profile of lambs supplemented with a polyherbal mixture ^1^ during the final fattening period.

Parameter	Treatment				EEM	*p*-Value	
	CON	HM1	HM2	HM3		Linear	Quadratic
Hematocrit, %	35.77	37.06	36.22	36.38	0.77	0.77	0.48
Hemoglobin, g dL^−1^	11.77	12.30	12.16	12.16	0.28	0.42	0.37
Red blood cells, 10^6^ mL^−1^	7.71	8.13	7.80	7.85	0.16	0.93	0.27
Mean corpuscular volume, fL	46.78	45.50	46.28	46.31	0.81	0.85	0.43
Mean corpuscular hemoglobin, pg	15.26	15.10	15.50	15.50	0.26	0.35	0.76
Mean corpuscular hemoglobinconcentration, g dL^−1^	32.83 ^b^	33.17 ^ab^	33.54 ^a^	33.57 ^a^	0.22	0.01	0.51
Platelets, 10^3^ mL^−1^	362.11	344.37	365.00	345.22	35.07	0.84	0.97
Leukocytes, 10^3^ mL^−1^	11.66	18.81	18.85	16.80	2.75	0.21	0.11
Lymphocytes, 10^3^ mL^−1^	24.44 ^b^	33.00 ^a^	32.77 ^a^	26.66 ^b^	2.33	0.54	0.004
Monocytes, 10^3^ mL^−1^	2.00	2.37	2.66	2.55	0.52	0.41	0.65
Segmented neutrophils, 10^3^ mL^−1^	68.22 ^a^	59.37 ^b^	58.33 ^b^	66.00 ^ab^	2.35	0.47	0.001
Band neutrophils, 10^3^ mL^−1^	3.11	3.25	3.88	2.33	0.52	0.47	0.12
Eosinophils, 10^3^ mL^−1^	1.77	1.75	2.00	2.44	0.53	0.35	0.66
Basophils, 10^3^ mL^−1^	0.44 *	0.25 *	0.33	0.00	0.14	0.06	0.63
Plasma protein, g dL^−1^	9.13	9.12	9.17	9.30	0.15	0.42	0.67

^1^ Animunin Powder^®^ based on *Solanum xanthocarpum*, *Hedychium spicatum*, *Curcuma longa*, *Piper longum* and *Ocimum sanctum*. CON—basal diet without polyherbal mixture (HM); HM1—basal diet + 1 g of HM kg^−1^ of DM; HM2—basal diet + 2 g of HM kg^−1^ of DM; HM3—basal diet + 3 g of HM kg^−1^ of DM. EEM—standard error of the treatment means; ^a, b, c^—means within a row with different subscripts differ when *p* ≤ 0.05; *—indicates a tendency.

**Table 6:** Blood biochemical parameters of lambs supplemented with a polyherbal mixture ^1^ during the final fattening period.

Parameter	Treatment				EEM	*p*-Value	
	CON	HM1	HM2	HM3		Linear	Quadratic
Glucose, mg dL^−1^	83.55	78.33	85.87	81.33	2.58	0.94	0.89
Urea, mg dL^−1^	19.55 *	24.00 *	23.75	21.88	1.81	0.41	0.09
Uric acid, mg dL^−1^	0.60 ^c^	0.78 ^ab^	0.92 ^a^	0.77 ^b^	0.07	0.04	0.03
Cholesterol, mg dL^−1^	39.11	36.00	40.87	40.11	1.77	0.32	0.51
Total protein, g dL^−1^	8.57	8.15	8.67	8.40	0.17	0.98	0.67
Albumin, g dL^−1^	4.70	4.48	4.73	4.62	0.16	0.98	0.77
Globulin, g dL^−1^	3.87	3.72	3.93	3.77	0.09	0.83	0.98
Albumin/globulin	1.25	1.19	1.25	1.22	0.05	0.89	0.82
Bilirubin, mg dL^−1^	0.25	0.31	0.26	0.28	0.04	0.79	0.74
Creatinine, mg dL^−1^	1.14 ^a^	0.91 ^b^	0.81 ^b^	0.77 ^b^	0.11	0.02	0.40
Alkaline phosphatase, UI dL^−1^	74.78	80.89	83.50	67.33	13.6	0.74	0.42
Lactate dehydrogenase, UI dL^−1^	163.22	137.56	161.12	137.22	26.9	0.65	0.97
Calcium, mg dL^−1^	9.26	8.70	8.95	8.97	0.24	0.58	0.25
Phosphorus, mg dL^−1^	4.68	4.57	4.41	4.63	0.15	0.62	0.28

^1^ Animunin Powder^®^ based on *Solanum xanthocarpum*, *Hedychium spicatum*, *Curcuma longa*, *Piper longum* and *Ocimum sanctum*. CON—d basal diet without polyherbal mixture (HM); HM1—basal diet + 1 g of HM kg^−1^ of DM; HM2—basal diet + 2 g of HM kg^−1^ of DM; HM3—basal diet + 3 g of HM kg^−1^ of DM. EEM—standard error of the treatment means; ^a, b, c^—means within a row with different subscripts differ when *p* ≤ 0.05; *—indicates a tendency.

## Data Availability

The data presented in this study are available on request from the corresponding author.
